# Pre-Clinical and Clinical Efficiency of Complexes of Oligoribonucleotides with D-Mannitol against Respiratory Viruses

**DOI:** 10.3390/pharmaceutics10020059

**Published:** 2018-05-19

**Authors:** Nataliia Melnichuk, Vladimir Zarubaev, Iaryna Iosyk, Mychaylo Andreychyn, Larisa Semernikova, Zenoviy Tkachuk

**Affiliations:** 1 Institute of Molecular Biology and Genetics, National Academy Science of Ukraine, 03680 Kyiv, Ukraine; natalia.melnichuk8@gmail.com (N.M.); larisasemernikova@gmail.com (L.S.); 2 Pasteur Institute of Epidemiology and Microbiology, 14 Mira str, 197101 St. Petersburg, Russia; zarubaev@gmail.com; 3 Department of Infectious Diseases and Epidemiology, I. Ya. Horbachevsky Ternopil State Medical University, 46002 Ternopil, Ukraine; josyk.jaryna@gmail.com (I.I.); andreychyn@tdmu.edu.ua (M.A.)

**Keywords:** acute respiratory infections, oligoribonucleotides-D-mannitol complexes

## Abstract

Oligoribonucleotides-D-mannitol (ORNs-D-M) complexes possess antiviral, anti-inflammatory, and immunomodulatory actions. The aim of the present study was to evaluated an antiviral effect of ORNs-D-M against parainfluenza virus type 3 (PIV3); influenza CA709, PR834; avian influenza virus H5N2 (AIV) in vitro by a TCID_50_; hemadsorption and neuraminidase activity assays; and clinical efficiency of ORNs-D-M in patients with acute respiratory infections (ARIs) of various etiologies by PCR assay and AmpliSens test systems. It was observed that ORNs-D-M have an antiviral activity against the influenza CA709, PR834, PIV3, and AIV in vitro. The injectable dosage form of ORNs-D-M was shown to have a stronger antiviral effect compared to capsule form. It was also detected that the injectable form of ORNs-D-M significantly reduced the neuraminidase activity of influenza PR834. A complex treatment of patients with ORNs-D-M had a positive effect on the course of the disease, it accelerated patients’ recovery. Treatment with ORNs-D-M caused eradication of adeno- and influenza viruses in patients with ARI. This drug contributed to significant decrease in duration of febrile period and cough. Comprehensive treatment with ORNs-D-M improved the disease clinical findings significantly. Collectively, these results suggested that ORNs-D-M may be used at co-infection with influenza and other respiratory viruses as a medical antiviral drug.

## 1. Introduction

Influenza and other acute respiratory infections (ARIs) are the most common human diseases and are nearly 70% of all infectious diseases. Up to 90% of the population suffer from one of these infections at least once a year. The current epidemic process is characterized by simultaneous circulation of different types of influenza viruses and annual outbreaks respiratory viruses during the winter and spring seasons; however, parainfluenza viruses—for example—are prevalent throughout the year [1,2,3,4].

ARIs are caused by viral or bacterial pathogens and can result in hospitalization and death, particularly among young children, older adults, and other high-risk groups [5,6]. Viruses are the leading causes of acute respiratory disease throughout the world. The most common viral etiologic agents of ARIs are respiratory syncytial virus (RSV), human metapneumovirus (MPV), influenza A and B viruses, parainfluenza viruses (PIV 1-2-3) and adenoviruses (AdV), human rhinoviruses (RV), human coronaviruses (CoV), enteroviruses (EnV), and human bocavirus (BoV) [7,8,9]. In addition, human polyomaviruses (KI, WU) have been detected in patients with respiratory infections [7,10]. Although ARIs have traditionally been thought to be caused by single viruses, an increasing number of reports have reported respiratory diseases occurring as dual or multiple virus infections [11,12,13,14,15,16]. There are suggestions that respiratory viral co-infections affect disease severity with some studies suggesting that dual and multiple infections increase the severity of respiratory disease [12,14,16], while dual or multiple infections may actually be protective [13].

The human PIV type 3 (PIV3) is common human respiratory tract pathogen, which causes ARI, pneumonia, and bronchiolitis. PIV3 is known to be an important community-acquired pneumonia pathogen in transplant recipients and immunosuppressed patients, is also an important pathogen in immunocompetent adults. In normal adult hosts, the clinical presentation of PIV 3 pneumonia may clinically closely mimic H1N1 pneumonia [17].

Currently, there are no approved vaccines for the prevention of most respiratory viral infections despite continual efforts in this field [18,19,20,21]. Medications cover almost all possible responses to the infectious process and often do not show the desired results [19,22], and the idea of fast elimination of a viral agent from the cells of infected body is still a topical issue [23]. The emergence of drug-resistant influenza variants has led to a decline in the efficacy such drugs as oseltamivir (Tamiflu), amantadine (Symmetrel), and rimantadine (Flumadine) [24,25,26]. Therefore, new therapeutics against respiratory viral infections with novel mechanisms of action and against new targets are urgently required. Moreover, co-infection with influenza and other respiratory viruses decreases the efficiency of some aforementioned drugs [27,28].

It is established that oligoribonucleotides-D-mannitol (ORNs-D-M) complexes (total yeast RNA modified with D-mannitol) possess an antiviral, anti-inflammatory, and immunomodulatory effects [29]. These complexes were registered under the commercial name ‘Nuclex’ in Ukraine. This medicine effects influenza virus surface antigens, depresses virus activity, and increases interferon production during infection of influenza virus A/Fort Monmouth/1/1947-mouse adapted (H1N1) (FM147) in vitro [30,31]. ORNs-D-M as an antiviral drug proved to be effective in fighting with many infectious diseases [32,33,34]. It was not previously used by patients who suffered from influenza or other ARIs. This study was performed to determine an antiviral efficiency of ORNs-D-M drug against different strains of the influenza viruses and PIV3 and the clinical efficiency of ORNs-D-M in patients with ARI of various etiologies.

## 2. Materials and Methods

### 2.1. Materials

ORNs-D-Mannitol (ORNs-D-M) complexes—total yeast RNA is modified with D-mannitol (D-M). ORNs-D-M was purchased from the subsidiary company Biosell, Kyiv, Ukraine. Influenza virus A/California/07/2009(H1N1)pdm09 (CA709), A/PuertoRico/08/1934(H1N1) (PR834), avian influenza virus A/mallard/Pennsylvania/10218/84 (H5N2), and PIV3 were obtained from National Virus Collection of the D.I. Ivanovsky Institute of Virology of Russian Academy of Medical Science (RAMS) (Moscow, Russia). Human epithelial type 2 (HEp2) and Madin–Darby canine kidney (MDCK) cells were obtained from Russian Cell Culture Collection of the RAMS. The HEp2 cells were cultured in 96-well plates to a monolayer state in MEM medium adding 10% of fetal bovine serum (FBS) for cell growth culture of the virus.

One-hundred and eight patients of young and middle age with influenza and others ARI were observed in the Clinic of Infectious Diseases, I. Ya. Horbachevsky Ternopil State Medical University. All procedures performed in studies involving human participants were in accordance with the ethical standards of the institutional and/or national research committee and with the 1964 Helsinki declaration and its later amendments or comparable ethical standards.

### 2.2. Methods

#### 2.2.1. ORNs-D-M Antiviral Activity against PIV-3

Both forms (injectable and capsule) of ORNs-D-M at concentrations of 5.0, 1.7, 0.5, and 0.05 mg/mL were studied. To make the capsule form, ORNs-D-M were dissolved in MEM medium, whereas—for injectable form—making ORNs-D-M were dissolved in MEM medium containing 20 mg/mL of arginine. ORNs-D-M capsule and injectable forms had the same concentration of ORNs-D-M. The HEp2 cells were infected with the PIV3 (3 lgID_50_). Into each well of 96-well plates with monolayer cells was added 20 µL of the virus. After an hour of incubation at 37 °C, 80 μL of MEM medium with appropriate ORNs-D-M concentration was added into the wells with infected cells and the medium was further incubated at 37 °C, 5% CO_2_, for 72 h. The level of destructive changes of the HEp2 was detected by a light microscope. The control was infected cells with the virus without any drug. All forms of the experiment were performed three times. The medium was removed from the experimental and control wells. After cells were washed withphosphate-buffered saline (PBS) and was added 20 μL/well of 2% of solution of guinea pig red blood cells (GPRBCs) for determine the degree of hemagglutinin (HA) activity using hemadsorption assay. Plates with HEp2 cells and GPRBCs were incubated for 30 min at 4 °C. After incubation Hep2 cells were washed with PBS twice and hemadsorption HEp2 cells were observed under a light microscope. To quantify hemadsorption activity, we determined the average number of GPRBCs adsorbed per ten HEp2 cells by analysis of at least 250 HEp2 cells per replicate culture. Then each removed mediums was diluted from 10° to 10^−7^ and 100 μL/well of the diluted medium was added in 96-well plate with a monolayer of the HEp2 cells (eight wells were infected with one dilution) and were further incubated at 37 °C, 5% CO_2_, for 72 h. After infectivity of the PIV3 was determined by a hemadsorption assay. The infectious titer was expressed as the number (decimal logarithm, lgID_50_) of the highest dilution factor that produced HEp2 cells hemadsorption activity reading.

#### 2.2.2. ORNs-D-M Antiviral Activity against Pandemic Influenza CA709 and AIV in Vitro

The compounds in appropriate concentrations were dissolved in MEM with 1 µg/mL trypsin and incubated with MDCK cells for 1 h at 36 °C. Each concentration of the compounds was tested in triplicate. The cell culture was washed twice with PBS and incubated with appropriate viruses (MOI 0.01) for 1 h. The monolayers were washed twice with PBS, and the same compound-containing medium was added. The plates were incubated for 48 h at 36 °C in the presence of 5% CO_2_. A virus titer in the supernatant was further determined by TCID_50_ assay [35] after cultivating of the virus in MDCK cells for 48 h at 36 °C in the presence of 5% CO_2_. To detect the virus, culture medium (0.1 mL) was transferred to the corresponding wells of round-bottom plates and mixed with 0.1 mL of 1% chicken erythrocytes. The results were checked after 1 h of incubation at room temperature. The 50% inhibiting concentration (IC_50_) and selectivity index (SI, the ratio of CC_50_ to IC_50_) of each compound were calculated from the data obtained. Rimantadine was used as a reference compound.

#### 2.2.3. Neuraminidase Activity Assay

The study of anti-neuraminidase activity of compounds was conducted by measurement of activity of neuraminidase in the reaction with fluorogenic substrate [36]. The injectable and capsule forms were made during analysis of ORNs-D-M antiviral activity against the PIV3. The 25 µL of both forms of ORNs-D-M at concentrations of 10.0, 3.2, 1.0, and 0.1 µM and the 25 µL of virus (5×) that were diluted in assay buffer (32.5 mM MES buffer (pH 6.0), 4 mM CaCl_2_) were mixed in 96-well plate. After, the plate with ORNs-D-M and virus was incubated for 30 min at 37 °C. Then, 0.2 mM substrate solution (4-methylumbelliferyl-α-D-*N*-acetylneuraminic acid) was added to the wells and plates were further incubated for 30 min at room temperature. Reaction was stopped with a stop solution (25% ethanol, 0.1 M glycine pH 10.7) followed by measurement of luminescence on multifunctional plate reader Victor 1440 (Ex λ 365 nm, Em λ 450 nm). Based on the data obtained, 50% inhibiting concentrations of the compound were calculated for each virus. Oseltamivir carboxylate in three-fold dilutions (1 mM–0.1 nM) was used as a reference compound.

#### 2.2.4. Clinical Assay of ORNs-D-M Efficiency

To determine the etiology in patients with ARI, nasal and oropharynx swabs were evaluated by a polymerase chain reaction (PCR) using the Rotor-Gene 6000 and AmpliSens test systems, which are registered in Ukraine. All the patients were divided into the two groups: the first group (main group) consisted of 54 individuals who took ORNs-D-M complex therapy; the second group (control group) of 54 patients received standard treatment (Arbidol as etiotropical drug). The groups were formed on a random basis and were chosen according to age, sex, etiology of the disease, epidemiological criteria, and clinical signs of the disease. The criteria of patients’ inclusion in the study were patients with influenza and other ARIs, from 18 to 70 years old and both sexes. The exclusion criteria were children under 18 and adults under 70 years old, presence of severe concomitant diseases, that could affect the course of the disease, burdened allergic history, and mild illness that did not require hospitalization. To determine a severity of the disease, the degree of increasing body temperature and its duration, severity and duration of intoxication syndrome (general weakness, headache, myalgia, body aches, nausea, vomiting), and catarrhal syndrome (throat irritation, hoarseness of voice, cold, coughing) were identified by the responsible doctor. Increasing body temperature to 37.5–38.9 °С during 3–4 days, clinically evident intoxication, and catarrhal manifestations pointed at an average severity of the disease. Conversely, a sharp increase in body temperature to 39–41 °С, clinically evident intoxication, severe myalgia, significant general weakness, and the development of pneumonia pointed at a severe disease. The patients’ clinical samples were collected for PCR assay at the beginning of hospitalization and during the period of an early reconvalescence (7–10th day of illness). The 500 mg of ORNs-D-M was administered four times a day to ingest after meals for 5–7 days. The treatment effectiveness was determined due to clinical data and results of PCR.

Statistical analysis was performed with Statistica 10 software package. The data were presented as mean M ± m). A two-way analysis of Student’s *t*-test, Spearman rank correlation analyses, Mann–Whitney–Wilcoxon and Wilcoxon signed-rank tests were used. The *p* < 0.05 was considered significant.

This study was a fragment of the integrated research work “Improvement of diagnostic, medical and preventive measures at widespread viral and parasitic diseases” (state registration number 0114U001387), Department of Infectious Diseases and Epidemiology, Dermatology and Venereology, I. Ya. Horbachevsky Ternopil State Medical University, Ministry of Healthcare of Ukraine (MH of Ukraine). It was approved by the expert problematic commission of MH and National Academy Medical Sciences (NAMS) of Ukraine “Infectious and Parasitic Diseases” 27 June 2013 (protocol no. 39). The bioethics Commission of the I. Ya. Horbachevsky Ternopil State Medical University did not detect the moral and ethical norms violations during the study (protocol no. 35/25.11.2015). ORNs-D-M medicine was registered in Ukraine and introduced into clinical practice (single-blind study), decree of MH of Ukraine no 752/30.04.2010 (more than two years before the beginning of our studies). Our study included comparison of clinical and virological activity of ORNs-D-M and Arbidol. The Arbidol was approved for clinical use (registration number no. 295/21.04.2009).

## 3. Results

### 3.1. Inhibition of PIV3 Activity by Capsule and Injection Forms of ORNs-D-M In Vitro

**Figure 1:**
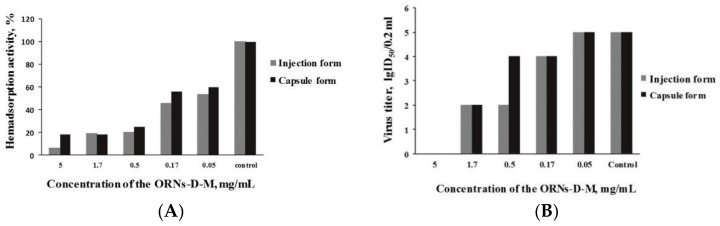
Inhibiting hemadsorption activity of the PIV3 (parainfluenza virus type 3) infected cells (**A**) and infectivity of the PIV3 (**B**) by the capsule and injection forms of oligoribonucleotides-D-mannitol complexes in vitro.

Antiviral effect of ORNs-D-M capsule and injection forms against the PIV3 in vitro was determined by their ability to inhibit a hemadsorption activity of HEp2 cells, that was infected with PIV3, and the PIV3 infectivity. The percentage of hemadsorption cells was from 6.2% to 20% after 72 h incubation with the PIV3 and the injection form of ORNs-D-M at concentrations from 5.0 to 0.5 mg/mL. When concentration of the injection form was decreased to 0.17 mg/mL, the number of cells absorbing red blood cells was 45.5%. However, 50 µg/mL of the injection form of the drug also inhibited the number of the hemadsorbtion cells by 55.4% (Figure 1). The capsule form of ORNs-D-M also significantly reduced hemadsorbtion ability of the PIV3 infected HEp2 cells. At concentrations from 5.0 to 0.5 mg/mL of ORNs-D-M capsule form, the percentage of hemadsorbtion cells ranged from 18.5% to 25% respectively, whereas hemadsorption activity of cells was reduced by 56.2% and 60.0% respectively after incubation with the drug at concentrations 0.17 and 0.05 mg/mL.

After 72 h incubation, PIV3 infected cells with the both forms of ORNs-D-M at concentration 5.0 mg/mL, infectivity of the PIV3 was inhibited. Both 1.7 and 0.5 mg/mL of ORNs-D-M injection form reduced the infectious titer of PIV3 by 3 IgID_50_ compared with the control, while only the 1.7 mg/mL of ORNs-D-M in capsule form decreased the infectious titer of PIV3 by 3 IgID_50_. Conversely, the infectious titer of PIV3—after incubation the virus with 0.17 mg/mL of the injection form and 0.5 and 0.17 mg/mL of the capsule form—decreased insignificantly and remained unchanged after incubation the virus with 0.05 mg/mL of the both forms in comparison to the control.

### 3.2. Antiviral Activity of ORNs-D-M against the Pandemic Influenza CA709 and AIV In Vitro

**Figure 2:**
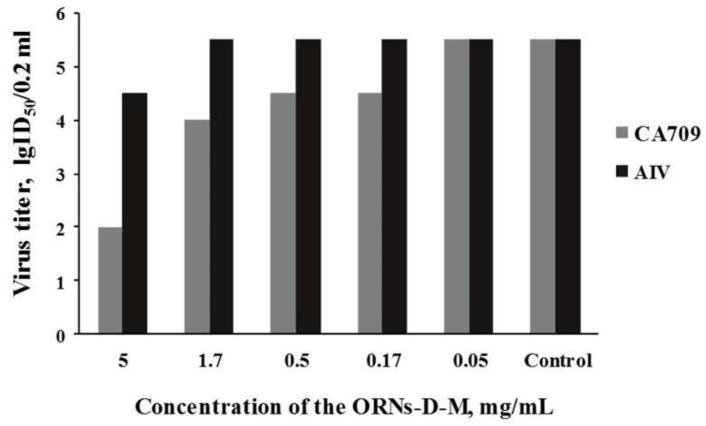
Antiviral activity of the oligoribonucleotides-D-mannitol complexes against the Influenza virus A/California/07/2009(H1N1)pdm09and avian influenza virus H5N2 in vitro.

It was also important to study antiviral effect of ORNs-D-M against other influenza virus strains, especially pandemic influenza CA709 and AIV. Effects of ORNs-D-M at different concentrations on infectivity of the pandemic influenza CA709 and AIV proved that the drug inhibited infectivity of these viruses on the НEр2 cells (Figure 2). So the effective dose of the drug against the pandemic virus CA709 was 0.5 mg/mL at selectivity index >10, and the acceptable concentration was 5.0 mg/mL. At concentration of 1.5 mg/mL, the inhibition of the virus strain was less effective by one-and-a-half logarithms. After drug concentration was decreased to 0.17 mg/mL, the effect remained within one logarithm. The drug concentration of less than 0.17 mg/mL had no anti-influenza effect. The results of this research also demonstrated that ORNs-D-M at a concentration of 5.0 mg/mL has activity against the AIV, selectivity index >3.

### 3.3. Antiviral Activity of the Capsule and Injection Forms of ORNs-D-M against the Influenza Virus PR834 In Vitro

**Figure 3:**
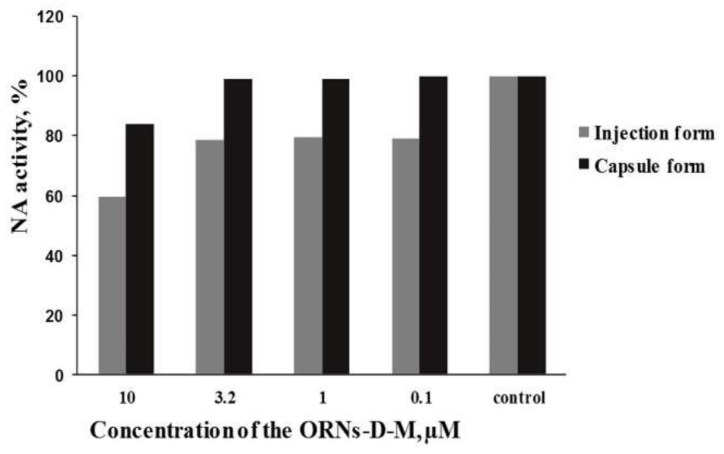
Level of the NA activity of influenza virus A/PuertoRico/08/1934(H1N1 under influence of the injection and capsule forms of oligoribonucleotides-D-mannitol complexes.

Inhibition of the NA activity of influenza virus PR834 by the both ORNs-D-M forms was studied (Figure 3). The rate of NA activity of the virus affected by ORNs-D-M injection form at a concentration of 10 μM reaches only 59.5%, and at concentrations of 3.2 and 1 μM the enzyme activity was ~80%. The lower concentration of the drug injection form had almost no effect. The drug capsule form had less effect on the NA activity of influenza PR834 at the specified concentrations. The drug had a positive effect at a concentration of 10 μM, where the rate of NA action was 83%.

### 3.4. Clinical Efficiency of ORNs-D-M

Severe course of the disease was observed in 28 (51.9%) patients of the main group and 21 (38.9%) patients of the control one. Moderate course was in 26 (48.1%) patients of the main group and 33 (61.1%) patients of the control group, without any significant difference between the groups (*p* > 0.05). Pneumonia as a complication was diagnosed in 25 (23%) patients: 10 (40%) patients of the main group and 15 (60%) of the control one (*p* > 0.05). Both groups of patients were the same by gender and age (*p* > 0.05). Main disease signs in the patients of both groups were: fever, intoxication, and catarrhal syndromes, which identified the severity of the disease.

The examination of the patients by PCR during their stay at hospital (during the first three days of illness) showed positive results in 78 (72.2%) patients of both groups. In six patients (7.7% with positive results) of both groups, various combinations of viruses were revealed: two pathogens simultaneously (double infection). The groups of patients, who were examined for disease etiology, did not differ significantly (*p* > 0.05).

A complex treatment with ORNs-D-M of the main group of patients had a positive effect on the course of the disease, it accelerated patients’ recovery. This drug contributed to a significant decrease in duration of febrile period. So, it lasted in average 1.8 ± 0.2 days in the main group and 2.6 ± 0.2 days in the control group, *p* < 0.001. Increased body temperature was observed only during the first two days in 85.1 ± 4.8% patients of the main group, it was longer only in 14.9 ± 4.8% persons, whereas in the control group, short-term (1–2 day) hyperthermia was recorded only in 51.8 ± 6.8% of patients, and in 48.2 ± 6.9% of them it lasted for three days or longer (*p* < 0.001). Dependence of etiological factor influence on duration of febrile period within the adapted treatment was not established. Cough lasted for two-three days in 59.3 ± 6.7% of patients of the main group, while the number of persons with cough was much lower in the control group 35.1 ± 6.5% (*p* < 0.01). Duration of cough for six days or longer in patients of the main group 29.6 ± 6.2 was significantly lower, (*p* < 0.001), if compared to the control one 55.5 ± 6.8%. On average, in the group treated with ORNs-D-M cough lasted for 3.8 ± 0.3 days and 5.9 ± 0.7 days in the control group of patients (*p* < 0.001).

Treatment with ORNs-D-M of the examined patients contributed not only to the regression of the disease clinical manifestations, but to the normalization of the number of laboratory findings as well. The dependence of the main symptoms’ duration and the disease severity in patients, who received different treatment, was defined (Table 1). Duration of intoxication and catarrhal syndromes and hospital stay in the patients of the main group with moderate ARI severity were shorter than in those with severe course of the disease (*p* < 0.05). The same correlation was in the patients of the control group. Statistically significant effect of ORNs-D-M in reducing the main clinical symptoms (in 1.5 times) and the need of in-patient treatment was denoted for both moderate and severe course groups (*p* < 0.05) (Table 1).

The influence of different treatment on duration of the disease main manifestations was studied in dependence of pneumonia presence in the patients. Data presented in Table 2 shows that fever and intoxication duration was considerably shorter in ORNs-D-M-treated patients than in the same group of patients with ARI uncomplicated or complicated with pneumonia (*p* < 0.05). Duration of cough in the main group was only 5.1 ± 0.4 days and 7.5 ± 0.8 days in the control group (*p* < 0.05). In patients who did not receive ORNs-D-M all clinical signs lasted longer and they stayed longer at hospital. The virus elimination depending on different treatment methods was studied. The etiology of 40 people of the main group was deciphered during the admission according to PCR (43 positive results). In early convalescence, pathogens were found again only in 3 (7.5%) of these patients of the main group and in 22 (57.9%) of 38 patients of the control group (*p* < 0.001).

In all cases of polyinfections, the results of PCR in early convalescence were negative. In the main group, influenza A virus eradication was reached in all positive cases, but in the control group the virus was detected in 9 of 16 patients, (*p* < 0.001). Eradication of RSV was evidenced in 13 of 14 patients (1 detected) of the main group and only in 5 of 13 patients of the control group (8 detected) (*p* < 0.01). The eradication of AdV was found in all patients of the main group and only in three of five (two detected) of the control group. The PIV was observed in two of eight patients of the main group and in four of seven patients of the control group, (*p* < 0.01). Therefore, due to PCR, the virus was found more rarely in the patients who were treated with ORNs-D-M (*p* < 0.001), Table 3.

## 4. Discussion

The oligoribonucleotides-D-mannitol (ORNs-D-M) complexes (total yeast RNA modified with D-mannitol (D-M)) have a wide range of biological activities and can be used in antiviral treatment, playing a key role in antiviral activity [29]. Viral pathogens are the most common etiological agents of ARIs [13,15,37]. In the presented study, ORNs-D-M were evaluated for their ability to inhibit the PIV3, pandemic influenza CA709, and influenza virus PR834 in vitro. The study of ORNs-D-M antiviral effect against the PIV3 in vitro proved that the both forms of the drug can inhibit this virus infectivity. The injection form of ORNs-D-M had stronger anti-PIV3 effect than the capsule form. The degree of antiviral effect of both forms depends on the concentration of the drug. Analyses of available avian influenza viruses circulating worldwide suggest that most viruses are susceptible to oseltamivir, peramivir, and zanamivir [38]. However, some evidence of antiviral resistance has been reported in HPAI Asian lineage AIV (cdc.gov/flu/avianflu/prevention.htm). Therefore, it is important to search for the influenza antiviral drugs with a wide spectrum of antiviral action. The results of this research (Figure 2) proved that ORNs-D-M at concentration of 5.0 mg/mL have activity against the AIV, selectivity index >3. The obtained results suggest that ORNs-D-M can probably be used as an antiviral drug against an AIV. Whilst we found that ORNs-D-M have antiviral activity against the pandemic virus CA709, Figure 2.

The influenza virus has two surface antigens—HA and NA—which are responsible for adsorption, penetration, and release of the virus from cells [39]. Previously, we proved that ORNs-D-M has specific antiviral effect due to its ability to inhibit the NA action of the influenza virus (FM147) in vitro [30]. After comparison of the results of previous research on NA action of the influenza virus (FM147) [30], we proved the effective inhibition of the receptor in both studies. The inhibition 40.5% of NA activity strain A/PR8/34 (H1N1) by injection form of ORNs-D-M takes place in concentration (Figure 3) that is 50 times less than in the research on the influenza virus (FM147). These results suggest greater efficiency of the injection form, as well as different sensitivities of different strains of NA to the inhibiting effect of ORNs-D-M. Our results also suggest antiviral activity in ORNs-D-M against the influenza CA709, PR834, AIV, and PIV3 in vitro. These antiviral activities of ORNs-D-M can be attributed to inhibiting the NA activity and HA–glycan interaction of influenza viruses [30,31] and inhibiting the Toll-like receptors 3, 7, and 8, impairing the upregulation of genes induced by viruses [40].

In our previous studies, we found that ORNs-D-M had clinical effects in treating patients with chronic hepatitis C and with diabetes type 2 [33,34]. In this study, the clinical data showed that the comprehensive treatment of the influenza A virus, PIV, RSV, AdV infections, and other ARIs (unidentified) with ORNs-D-M improved the disease clinical findings significantly; decreased the duration of fever, intoxication syndrome, coughing, and hospital stay duration of patients with moderate and severe forms of disease. Treatment with ORNs-D-M caused eradication of the influenza A virus, AdVviruses in patients with the influenza and other ARIs.

## 5. Conclusions

Our experimental and clinical results show that ORNs-D-M has antiviral activity against a wide range spectrum of respiratory viruses. The results obtained indicate that ORNs-D-M can be used for treatment co-infection with influenza and other respiratory viruses. The injectable dosage form of ORNs-D-M has a stronger antiviral effect compared to the capsule form. In future research, pre-clinical (against AIV, influenza CA709) and clinical efficiency of ORNs-D-M injection form should be studied.

## Figures and Tables

**Table 1:** Duration of main syndromes in the patients with ARI (Influenza A virus, PIV, AdV, RSV) of different severity and duration of hospital stay due to the treatment method and disease course (days, M ± m).

Syndromes	Main Group		Control Group	
	Severity of Disease			
	Moderate, *n* = 26	Severe, *n* = 28	Moderate, *n* = 33	Severe, *n* = 21
Fever	1.9 ± 0.1	2.9 ± 0.2 ***	2.4 ± 0.2 *	3.4 ± 0.2 **^,^***
Intoxication	1.8 ± 0.2	2.6 ± 0.2 ***	2.6 ± 0.2 *	3.9 ± 0.5 **^,^***
Catarrhal	2.9 ± 0.3	4.4 ± 0.5 ***	4.4 ± 0.3 *	8.6 ± 0.9 **^,^***
Duration of hospitalization	7.8 ± 0.3	9.4 ± 1.8 ***	9.9 ± 0.6 *	14.7 ± 1.9 **^,^***

*—differences (*р* < 0.05) in moderate form of disease; **—in groups with severe forms; ***—between groups with moderate and severe forms of disease.

**Table 2:** Duration of main syndromes in patients with ARI (Influenza A virus, PIV, AdV, RSV) due to the pneumonia presence (days, M ± m).

Syndromes	Main Group		Control Group	
	ARI, *n* = 44	ARI and Pneumonia, *n* = 10	ARI, *n* = 39	ARI and Pneumonia, *n* = 15
Fever	1.9 ± 0.2	2.8 ± 0.5 *	2.3 ± 0.2 **	4.2 ± 0.3 *^,^**
Intoxication	1.9 ± 0.1	3.0 ± 0.3 *	2.6 ± 0.2 **	4.3 ± 0.6 *^,^**
Catarrhal	3.6 ± 0.3	5.1 ± 0.4 *	4.1 ± 0.3 **	7.5 ± 0.8 *^,^**
Duration of hospital stay	8.1 ± 0.4	12.9 ± 0.9 *	10.3 ± 0.6 **	17.2 ± 1.8 *^,^**

*—differences (*р* < 0.05) between complicated and uncomplicated ARI of the same group; **—between the groups.

**Table 3:** Virus frequency in patients with ARI (Influenza A virus, PIV, AdV, RSV) before and after treatment according to PCR.

Viruses	Main Group		Control Group	
	(*n* = 40)		(*n* = 38)	
	Before Treatment	After Treatment	Before Treatment	After Treatment
Influenza A virus	16	0	9	6
PIV	8	2	13	4
AdV	5	0	5	2
RSV	14	1	13	9

## References

[1] Bisno A.L. (2001). Acute pharyngitis. N. Engl. J. Med..

[2] Scholtissek C. (1995). Molecular evolution of influenza viruses. Virus Genes.

[3] Verhagen J., Sander H., Fouchier Ron A.M. (2015). Infectious disease: How a virus travels the world. Science.

[4] Ghedin E., Fitch A., Boyne A., Griesemer S., DePasse J. (2009). Mixed infection and the genesis of influenza virus diversity. J. Virol..

[5] Tsalik E.L., Henao R., Nichols M. (2016). Host gene expression classifiers diagnose acute respiratory illness etiology. Sci. Transl. Med..

[6] Talbot H.K., Falsey A.R. (2010). The diagnosis of viral respiratory disease in older adults. Clin. Infect. Dis..

[7] Sloots T.P., Whiley D.M., Lambert S.B. (2008). Emerging respiratory agents: New viruses for old diseases?. J. Clin. Virol..

[8] Jacques J., Moret H., Minette D. (2008). Epidemiological, molecular, and clinical features of enterovirus respiratory infections in French children between 1999 and 2005. J. Clin. Microbiol..

[9] Weiss S.R., Navas-Martin S. (2005). Coronavirus pathogenesis and the emerging pathogen severe acute respiratory syndrome coronavirus. Microbiol. Mol. Biol..

[10] Kahn J.S. (2007). Newly discovered respiratory viruses: Significance and implications. Curr. Opin. Pharmacol..

[11] Esper F.P., Spahlinger T., Zhou L. (2011). Rate and influence of respiratory virus co-infection on pandemic (H1N1) influenza disease. J. Infect..

[12] Cilla G., Onate E., Perez-Yarza E.G. (2008). Viruses in community acquired pneumonia in children aged less than 3 years old: High rate of viral coinfection. J. Med. Virol..

[13] Martin E.T., Kuypers J., Wald A. (2012). Multiple versus single virus respiratory infections: Viral load and clinical disease severity in hospitalized children. Influenza Other Respir. Viruses.

[14] Drews A.L., Atmar R.L., Glezen W.P. (1997). Dual respiratory virus infections. Clin. Infect. Dis..

[15] Portnoy B.E., Eckert H.L., Hanes B. (1965). Multiple respiratory virus infections in hospitalised children. Am. J. Epidemiol..

[16] Semple M.G., Cowell A., Dove W. (2005). Dual infection of infants by human metapneumovirus and human respiratory syncytial virus is strongly associated with severe bronchiolitis. J. Infect. Dis..

[17] Cunha B.A., Corbett M., Mickail N. (2011). Human parainfluenza virus type 3 (HPIV 3) viral community-acquired pneumonia (CAP) mimicking swine influenza (H1N1) during the swine flu pandemic. Heart Lung.

[18] Nichols W.G., Corey L., Gooley T. (2001). Parainfluenza virus infections after hematopoietic stem cell transplantation: Risk factors, response to antiviral therapy, and effect on transplant outcome. Blood.

[19] Abed Y., Boivin G. (2006). Treatment of respiratory virus infections. Antivir. Res..

[20] Davlin S.L. (2016). Influenza activity—United States, 2015–2016 season and composition of the 2016–2017 influenza vaccine. Morb. Mortal. Wkly. Rep..

[21] Houser K., Subbarao K. (2015). Influenza vaccines: Challenges and solutions. Cell Host Microbe..

[22] El-Sahly H. (2000). Spectrum of clinical illness in hospitalized patients with “common cold” virus infections. Clin. Infect. Dis..

[23] Jain S., Kamimoto L., Bramley A.M. (2009). Hospitalized Patients with H1N1 Influenza in the United States, April–June 2009. N. Engl. J. Med..

[24] Bright R.A., Shay D.K., Shu B. (2006). Adamantane resistance among influenza A viruses isolated early during the 2005–2006 influenza season in the United States. JAMA.

[25] Moscona A. (2009). Global transmission of oseltamivir-resistant influenza. N. Engl. J. Med..

[26] Sheu T.G. (2011). Dual resistance to adamantanes and oseltamivir among seasonal influenza A (H1N1) viruses: 2008–2010. J. Infect. Dis..

[27] Matthew P., Hall R.J., Sonnberg S. (2010). Pandemic (H1N1) 2009 and Seasonal Influenza A (H1N1) Co-infection, New Zealand, 2009. Emerg. Infect. Dis..

[28] Chertow D.S., Memoli M.J. (2013). Bacterial coinfection in Influenza A. JAMA.

[29] Tkachuk Z. (2013). Multiantivirus Compound, Composition and Method for Treatment of Virus Diseases. U.S. Patent.

[30] Tkachuk Z.Y., Rybalko S.L., Zharkova L.D. (2010). Antiinfluenzal activity of drug Nuclex. Rep. Natl. Acad. Sci. Ukr..

[31] Melnichuk N., Semernikova L., Tkachuk Z. (2017). Complexes of Oligoribonucleotides with d-Mannitol Inhibit Hemagglutinin–Glycan Interaction and Suppress Influenza A Virus H1N1 (A/FM/1/47) Infectivity In Vitro. Pharmaceuticals.

[32] Tkachuk Z. (2011). Antiherpetic drug Nuclex effect. Rep. Natl. Acad. Sci. Ukr..

[33] Tkachuk Z.Y., Frolov V.M., Sotska Y.A., Kruglova O.V. (2012). Nuclex therapy for patients with chronic hepatitis C. Int. J. Immunol. Stud..

[34] Zelyoniy I.I., Tkachuk Z.Y., Afonin D.N., Tiutiunnyk A.A. (2013). Influence of Preparation Nucleх on the Cytokine Profile of the Patients with Diabetes Type 2 and Neuropathic Form of Diabetic Foot. J. Diabetes Res..

[35] Reed L.J., Muench H. (1938). A simple method of estimating fifty percent endpoints. Am. J. Hyg..

[36] Potier M., Belislem M., Dallaire L., Melanxson S.B. (1979). Fluorometric assay of neurominidase with a sodium (4-methylumbelliferyl-a-d-*N* ctylneuraminate) substrate. Anal. Biochem..

[37] Bonzel L., Tenenbaum T., Schroten H. (2008). Frequent detection of viral coinfection in children hospitalized with acute respiratory tract infection using a real-time polymerase chain reaction. Ped. Infect. Dis. J..

[38] Beigel J., Bray M. (2008). Current and future antiviral therapy of severe seasonal and avian influenza. Antivir. Res..

[39] Nayak D.P., Hui E.K., Barman S. (2004). Assembly and budding of influenza virus. Virus Res..

[40] Melnichuk N., Tkachuk Z. Influence of the oligoribonucleotides-d-mannitol complexes on upexpression of some genes induced by influenza virus in vivo. Proceedings of the 3rd International Electronic Conference on Medicinal Chemistry MDPI AG.

